# Delayed Pulmonary Manifestations of Miliary Tuberculosis Following a Normal Initial High-Resolution Computed Tomography

**DOI:** 10.7759/cureus.77098

**Published:** 2025-01-07

**Authors:** Yasushi Murakami, Shun Shibata, Mika Morosawa, Yasuhiro Nozaki, Yoshio Takesue

**Affiliations:** 1 Department of Respiratory Medicine, Tokoname City Hospital, Tokoname, JPN; 2 Department of Neurology, Tokoname City Hospital, Tokoname, JPN; 3 Department of Clinical Infectious Diseases, Tokoname City Hospital, Tokoname, JPN

**Keywords:** blood culture, bone marrow aspiration, fever of unknown origin, high-resolution computed tomography, miliary tuberculosis

## Abstract

Miliary tuberculosis (TB) is a potentially fatal form of TB resulting from the widespread dissemination of *Mycobacterium tuberculosis*. Although the presence of pulmonary miliary infiltrates usually facilitates diagnosis, atypical cases lacking these characteristic imaging findings frequently confound clinicians. Here, we describe the case of an 82-year-oldJapanese woman with miliary TB who initially presented with fever but showed no abnormalities on high-resolution computed tomography (HRCT) and microbiological tests; hence, fever of unknown origin was diagnosed. The delayed appearance of miliary infiltrates on repeated HRCT and positive interferon-gamma release assay (IGRA) led to a definitive diagnosis through bone marrow aspiration, bronchoalveolar lavage, and blood cultures. This case highlights the importance of miliary TB as a differential diagnosis for persistent fever, although initial imaging studies showed no abnormalities. Additionally, we discuss the value of repeated HRCT, IGRA, and minimally invasive diagnostic procedures for the early detection and timely treatment of miliary TB.

## Introduction

Miliary tuberculosis (TB) is a disseminated form of TB associated with high morbidity and mortality [1]. Miliary TB diagnosis is typically established by identifying characteristic pulmonary miliary infiltrates or detecting evidence of *Mycobacterium tuberculosis* infection in multiple organs [1,2]. Historically, many cases of miliary TB were diagnosed based solely on bacteriology or on clinical and radiographic findings, making antemortem diagnosis often challenging. However, with the advent of high-resolution computed tomography (HRCT), diagnostic accuracy has substantially improved [1-3]. Nevertheless, the absence of typical radiographic imaging findings sometimes can complicate diagnosis, particularly in uncommon cases without HRCT abnormalities [1,2,4]. Here, we describe the case of a patient with miliary TB who presented with fever but showed no abnormalities on initial HRCT and microbiological tests; thus, fever of unknown origin (FUO) was diagnosed. Subsequent development of miliary pulmonary infiltrates on repeated HRCT and positive interferon-gamma release assay (IGRA) served as key indicators for the final diagnosis.

## Case presentation

An 82-year-old Japanese woman residing in a long-term care facility presented to our hospital for the evaluation of a persistent fever lasting 12 days. She was treated with oral antibiotics for a suspected urinary tract infection (UTI) without improvement. The patient's medical history included dementia, depression, and stroke, with no history of immunosuppressive disorder or TB. Regular medications included antiplatelet agents and antidepressants. Upon arrival, communication was challenging because of dementia; however, no significant changes in the patient's mental status were observed compared to that at baseline. Although previously wheelchair-bound, her activity level had declined to bedridden status upon presentation. The patient had no complaints of pain, respiratory symptoms, changes in bowel habits, or other gastrointestinal symptoms. Her body weight was 36.3 kg (body mass index: 15.5 kg/m²) and vital signs were as follows: blood pressure, 108/68 mmHg; body temperature, 37.8°C; heart rate, 68 bpm; respiratory rate, 19 breaths/min; and oxygen saturation, 94% on ambient air.

**Figure 1:**
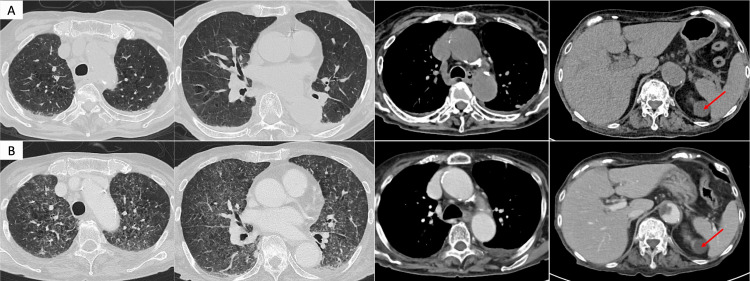
CT findings during hospitalization. (A, B) Non-contrast CT on day 0 (admission) and contrast-enhanced CT on day 13, respectively. (A) There were no abnormal findings in the lung parenchyma except passive atelectasis in the dependent areas. No hepatomegaly or splenomegaly is observed. A round lesion adjacent to the spleen is identified as a left-sided renal cyst (arrow). (B) Widespread, poorly defined small nodules and ground-glass opacities are observed diffusely in both lungs. The liver and spleen exhibited no morphological abnormalities or enlargements. A left renal cyst is observed (arrow). CT: computed tomography

Physical examination revealed no neck stiffness, oral abnormalities, superficial lymphadenopathy, or skin rashes. Laboratory findings included anemia (hemoglobin: 9.3 mg/dL), hypoalbuminemia (1.5 g/dL), hypokalemia (2.9 mmol/L), and elevated C-reactive protein level (2.54 mg/dL). Urinalysis showed no pyuria. HRCT revealed no abnormalities, excluding emphysema and passive atelectasis in the dependent areas, and there was no evidence of lymphadenopathy, hepatosplenomegaly, pleural effusion, or ascites (Figure 1A). Antibiotic treatment for presumed UTI was continued with intravenous ampicillin-sulbactam for five days but was ineffective. The patient met the classic diagnostic criteria for FUO [5], prompting further investigation to identify its underlying cause.

**Figure 2:**
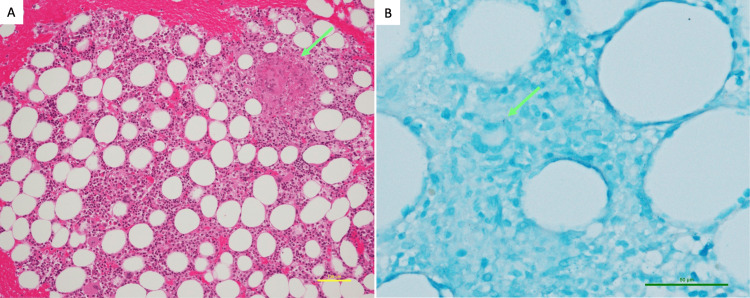
Pathological findings of bone marrow aspirate. (A) Granuloma formation with Langhans giant cells is observed (arrow). The bone marrow shows normal trilineage hematopoiesis. (B) Acid-fast bacilli are identified within the granuloma. Staining: (A) hematoxylin and eosin staining and (B) Ziehl-Neelsen staining. Scale bar: (A) 100 μm and (B) 50 μm.

Urine and blood cultures performed within the first week of hospitalization yielded negative results. Key laboratory test results in the work-up for FUO are summarized in Table 1. Neither hypogammaglobulinemia nor human immunodeficiency virus infection was detected. Elevated soluble interleukin-2 receptor (sIL-2R) levels (3940 U/mL) raised the suspicion of malignant lymphoma. Although the IGRA performed on day 7 was positive, it was initially considered a latent TB infection rather than an active disease. Because sIL-2R levels were markedly elevated, we suspected malignant lymphoma and therefore performed bone marrow aspiration (BMA) on day 12 and contrast-enhanced CT on day 13. While neither examination revealed findings suggestive of malignancy or abscesses, the repeated CT demonstrated new, diffuse, small nodular opacities in the lungs (Figure 1B). Based on positive IGRA and HRCT findings, we suspected disseminated TB and performed culture tests for *Mycobacterium* using bronchoalveolar lavage fluid (BALF), sputum, urine, and blood samples. Owing to concurrent antiplatelet therapy, transbronchial biopsy (TBB) and liver biopsy were deferred. As the patient's general condition progressively deteriorated with persistent high fever, we initiated empirical anti-TB therapy on day 15 with isoniazid (200 mg/day), rifampicin (300 mg/day), and ethambutol (500 mg/day), avoiding pyrazinamide due to concerns about a risk of drug-induced hepatotoxicity. Polymerase chain reaction tests for *M. tuberculosis* yielded negative results for all specimens. The patient's high fever and clinical condition gradually improved, allowing discharge on day 61. *M. tuberculosis* susceptible to first-line anti-TB drugs was isolated from both BALF and blood cultures, confirming the diagnosis of disseminated TB. Although the BMA culture was negative, histopathological examination revealed granuloma formation with Langhans giant cells and acid-fast bacilli (Figure 2).

**Table 1:** Summary of laboratory test results in the work-up for fever of unknown origin. Since multiple tests were conducted, only the initial results are presented. Complete blood counts and biochemical data are obtained upon admission. WBC: white blood cells; RBC: red blood cells; Hgb: hemoglobin; PLT: platelets; IgG: immunoglobulin G; IgA: immunoglobulin A; IgM: immunoglobulin M; MPO-ANCA: myeloperoxidase-specific antineutrophil cytoplasmic antibody; sIL-2R: soluble interleukin-2 receptor; TP: total protein; Alb: albumin; AST: aspartate aminotransferase; ALT: alanine aminotransferase; LDH: lactate dehydrogenase; ALP: alkaline phosphatase; γ-GTP: gamma-glutamyl transpeptidase; T-Bil: total bilirubin; BUN: blood urea nitrogen; Cr: creatinine; CRP: C-reactive protein; PCT: procalcitonin; HbA1c: glycated hemoglobin

Parameter		Result		Normal range
WBC		6	×10^3^/mm^3^	4-9
Neutrophils		76.2	%	40-60
Lymphocytes		13.4	%	20-40
RBC		3.4	×10^6^/mm^3^	3.5-5
Hgb		9.3	g/dL	11-15
PLT		155	×10^3^/mm^3^	150-350
Ferritin		689	ng/mL	6-138
IgG		1712	mg/dL	680-1620
IgA		410	mg/dL	84-438
IgM		84	mg/dL	57-288
MPO-ANCA		<1	U/mL	0-3.5
sIL-2R		3940	U/mL	157-474
TP		4.5	g/dL	6.6-8.1
Alb		1.5	g/dL	4.1-5.1
AST		34	IU/L	13-30
ALT		21	IU/L	7-23
LDH		277	IU/L	135-214
ALP		128	IU/L	35-104
γ-GTP		17	IU/L	9-32
T-Bil		0.2	mg/dL	0.2-1.2
BUN		23.2	mg/dL	8-20
Cr		0.87	mg/dL	0.46-0.79
Sodium		138	mmol/L	138-146
Potassium		2.9	mmol/L	3.6-4.9
Chloride		101	mmol/L	99-109
CRP		2.54	mg/dL	0-0.3
PCT		0.29	ng/mL	0-0.5
HbA1c		5.6	%	4.6-6.2

## Discussion

Presently, the absence of abnormalities in the initial imaging and microbiological studies made the diagnostic process particularly challenging. The delayed appearance of miliary shadows on the lungs and a positive IGRA result raised the suspicion of disseminated TB, ultimately leading to a diagnosis through BMA, bronchoalveolar lavage (BAL), and blood cultures. This case highlights several key points in the management of miliary TB. Although HRCT is performed, pulmonary lesions may not be detectable in the early stages, and repeated HRCT can be valuable in such cases. Diagnostic procedures, such as blood culture, BMA, and BAL, are effective and feasible for confirming the diagnosis, even in elderly patients at a high risk of bleeding.

Miliary TB was originally defined based on pathological findings of millet-sized granulomas and their corresponding radiographic features [1,2]. Currently, miliary TB encompasses all forms of disseminated TB resulting from the lymphohematogenous spread of *M. tuberculosis *[6]. This condition predominantly affects vulnerable populations, such as the elderly and immunocompromised patients. Provided its high mortality rate (25-30%), prompt diagnosis and timely treatment are crucial [1]. The clinical manifestations of miliary TB are diverse and nonspecific, necessitating a three-step diagnostic approach: (1) suspecting miliary TB, (2) estimating the involved organs based on clinical and imaging findings, and (3) obtaining samples from these organs for culture or pathological examination.

Although Japan is not classified as a TB-endemic country, in regions with a high TB burden, miliary TB should be considered regardless of the patient's immune status or presenting symptoms [7]. Imaging findings and IGRA are useful diagnostic tests for estimating the likelihood of TB. However, the limitations of IGRA (decreased sensitivity in immunocompromised individuals and inability to distinguish between latent and active infections) should be understood [2,8]. Here, positive IGRA during the diagnostic work-up of FUO prompted the reconsideration of TB as a potential diagnosis.

The lungs are the most commonly affected organs in patients with miliary TB, followed by the lymph nodes, liver, spleen, and bone marrow [2]. If initial evaluations reveal miliary shadows, lymphadenopathy, or hepatosplenomegaly, identification of the involved organs is straightforward. Presently, despite thorough evaluations by a radiologist and a respiratory physician, the initial HRCT revealed no abnormalities compared with previous imaging studies. Abnormalities in liver function tests and blood cell counts can serve as markers to estimate the involvement of the liver or bone marrow [9,10]; however, in this case, only minor abnormalities were detected. A significant aspect of our case was the delayed appearance of miliary infiltrates following an initially negative HRCT result. In a retrospective study involving 117 patients with miliary TB, initial HRCT failed to detect the disease in 13 cases (11%), and ill-defined nodules <2 mm were identified as a risk factor for missed diagnoses [4]. Among these missed cases, five underwent repeat CT scans within two months, subsequently confirming the emergence of miliary shadows. These findings suggest that if miliary TB is suspected, although the initial HRCT result is negative, repeating the HRCT may be useful for identifying the involved organs.

The diagnosis of miliary TB is established by identifying *M. tuberculosis* or caseating granulomatous inflammation in samples obtained from infected organs. Diagnostic procedures targeting clearly infected organs (e.g., TBB, lymph node biopsy, or liver biopsy) provide acceptable diagnostic yield [2]. If the involved organs are unclear, testing samples from the liver, bone marrow, or blood are useful, with reported sensitivities of 91-100%, 50-93%, and 14-30%, respectively [2]. Particularly, BMA and blood cultures are minimally invasive and carry a low bleeding risk, making them useful as screening tests [2,11]. In this case, owing to the patient's advanced age, poor general condition, and increased bleeding risk from antiplatelet therapy, only low-risk procedures were prioritized. Consequently, a definitive diagnosis was made based on BAL, BMA, and blood cultures. As culture and pathological examinations can take several weeks to obtain results, initiating anti-TB therapy without waiting for results, as demonstrated in our case, is important to improve patient outcomes [12].

## Conclusions

Miliary TB can present without detectable abnormalities on initial HRCT, leading to diagnostic challenges and delayed treatment. Our case highlights the importance of considering miliary TB in the differential diagnosis of FUO despite unremarkable initial imaging and microbiological studies. Repeated HRCT scans may reveal delayed pulmonary manifestations, thereby enhancing diagnostic accuracy. Clinicians should recognize these findings and develop effective strategies to diagnose and treat this potentially fatal condition promptly.
